# Feature group partitioning: an approach for depression severity prediction with class balancing using machine learning algorithms

**DOI:** 10.1186/s12874-024-02249-8

**Published:** 2024-06-03

**Authors:** Tumpa Rani Shaha, Momotaz Begum, Jia Uddin, Vanessa Yélamos Torres, Josep Alemany Iturriaga, Imran Ashraf, Md. Abdus Samad

**Affiliations:** 1 Department of Computer Science and Engineering, Dhaka University of Engineering & Technology, Gazipur, 1707 Bangladesh; 2 Department of Computer Science and Engineering, Bangabandhu Sheikh Mujibur Rahman Science & Technology University, Gopalganj, 8100 Bangladesh; 3 AI and Big Data Department, Woosong University, Daejeon, 34606 South Korea; 4 Universidad Europea del Atlántico, Santander, 39011 Spain; 5 Universidad Internacional Iberoamericana Campeche, Campeche, 24560 México; 6 Universidad de La Romana, La Romana, República Dominicana; 7 Universidad Internacional Iberoamericana Arecibo, Puerto Rico, 00613 USA; 8 Universidade Internacional do Cuanza, Cuito, Bié Angola; 9 Department of Information and Communication Engineering, Yeungnam University, Gyeongsan, 38541 South Korea

**Keywords:** Machine learning, Depression prediction, Class balancing, Oversampling, SMOTE, ADASYN, Stratified cross validation, Burn depression checklist, Feature group partitioning

## Abstract

In contemporary society, depression has emerged as a prominent mental disorder that exhibits exponential growth and exerts a substantial influence on premature mortality. Although numerous research applied machine learning methods to forecast signs of depression. Nevertheless, only a limited number of research have taken into account the severity level as a multiclass variable. Besides, maintaining the equality of data distribution among all the classes rarely happens in practical communities. So, the inevitable class imbalance for multiple variables is considered a substantial challenge in this domain. Furthermore, this research emphasizes the significance of addressing class imbalance issues in the context of multiple classes. We introduced a new approach Feature group partitioning (FGP) in the data preprocessing phase which effectively reduces the dimensionality of features to a minimum. This study utilized synthetic oversampling techniques, specifically Synthetic Minority Over-sampling Technique (SMOTE) and Adaptive Synthetic (ADASYN), for class balancing. The dataset used in this research was collected from university students by administering the Burn Depression Checklist (BDC). For methodological modifications, we implemented heterogeneous ensemble learning stacking, homogeneous ensemble bagging, and five distinct supervised machine learning algorithms. The issue of overfitting was mitigated by evaluating the accuracy of the training, validation, and testing datasets. To justify the effectiveness of the prediction models, balanced accuracy, sensitivity, specificity, precision, and f1-score indices are used. Overall, comprehensive analysis demonstrates the discrimination between the Conventional Depression Screening (CDS) and FGP approach. In summary, the results show that the stacking classifier for FGP with SMOTE approach yields the highest balanced accuracy, with a rate of 92.81%. The empirical evidence has demonstrated that the FGP approach, when combined with the SMOTE, able to produce better performance in predicting the severity of depression. Most importantly the optimization of the training time of the FGP approach for all of the classifiers is a significant achievement of this research.

## Introduction

At this moment, depression possesses a greater prevalence than any other mental ailment on the globe [1]. Depression is a mental illness that is manifested by a pervasive feeling of melancholy and emptiness, as well as a loss of enthusiasm or enjoyment in things that were previously gratifying. It is susceptible for everybody, regardless of age, gender, or socioeconomic status. A confluence of genetic, biochemical, environmental, and psychological elements contributed to its development. According to WHO, 450 million people around the world are impacted by depression disorder and this number will rise to about 15% by 2030 [2]. As a major effect, depression ruins a person’s relationship with their loved ones, including their family, friends, and partners. This occurs because the individual suffering from depression departs from social engagements and finds it difficult to express themselves properly. Another consequence, it harms a person’s physical health due to the significant correlations that exist between depression and physical ailments [3]. Numerous studies pointed out the associations between depressive disorders and other medical conditions, such as cardiovascular disease, diabetes, stroke, respiratory disease, cancer, and obesity [3, 4].

According to the findings of many research, there appears a relationship between alcohol use disorder (AUD) and depressive illnesses [5]. Many individuals, to alleviate the symptoms of depression, seek solace in alcohol or drugs, which can eventually develop into misuse and dependence on these substances. In general, depression raised the probability of suicide ideation and led to a significant rise in the number of premature deaths. In addition, people who suffer from depression have a suicide risk that is twenty times greater than the ordinary population [6]. According to the statistics, every year approximately 800,000 fatalities occur because of depression disorder, and this tendency is larger among young people or students who live in nations with poor or intermediate incomes [7]. Overall, depression can drastically lower an individual’s quality of life, making it difficult for the individual to feel joy, happiness, and an overwhelming sense of purpose in their everyday activities. If treatment for this prior disease is delayed for an extended period, it can also create considerable problems for society and the gross economy [8]. Based on the findings of a previous study [9], it was found that persistent depression affects 54.3% of the population in Bangladesh. This staggeringly high prevalence of depression poses a grave concern for society. It is prominent that students have a much greater prevalence of depression than the general population [10].

Furthermore, depression is the most prevalent reason for students in Bangladesh to commit suicide. According to the findings of [11] the rate of suicide in this region is 39.6 fatalities per 1,000,000 inhabitants. It is important to keep awareness that depression is a curable disorder, and anybody who suffering from these symptoms needs to seek professional assistance. Furthermore, the job of diagnosing depression is often challenging since it necessitates extensive psychological testing by skilled psychiatrists at an early stage [12] as well as interviews, questionnaires, self-reports, or evidence from relatives and close companions. Nevertheless, it is typical for patients with depression to delay seeking medical attention until the condition has worsened [13]. So, the main motivation of this research is to accurately determine depression severity at an early stage to give effective counseling and rehabilitation.

Machine learning algorithms are in high demand as a means of inferring meaningful patterns from raw data, owing to the exponential growth of available digital information. Machine learning algorithms have seen extensive usage in the health and medical fields, but have seen far less adoption in the psychology and behavioral sciences. Researchers in the field of psychological analysis are increasingly gravitating toward the use of machine learning from statistical inferences [14]. Consequently, it is frequently utilized as a strong approach for sorting through enormous volumes of healthcare data [15]. Numerous review articles have recommended that machine learning algorithms including random forest (RF), decision tree (DT), support vector machine (SVM), naive base (NB), and k-nearest neighbor (KNN) be used to predict depression severity [16–18]. Simultaneously these techniques are becoming increasingly popular for their ability to anticipate the possibility of mental health issues among students [19]. This study primarily emphasizes developing a machine learning based model to predict the severity of depression with high accuracy.

Moreover, a pivotal obstacle in the field of machine learning is the attainment of the necessary level of classification accuracy when confronted with datasets that exhibit substantial disparities in class distributions [20]. The term “imbalanced data” is used to describe a dataset in which certain classes contain a significantly larger number of samples compared to others [21]. The class that occurs most frequently is commonly referred to as the majority class, and the class that occurs least frequently is known as the minority class [22]. It tends to exhibit a bias towards the majority class. Consequently, the infrequent events are often overlooked, even if the prediction model achieves a high overall precision [23]. The objective of this study is to improve the predicted accuracy by addressing the issue of class imbalance through the utilization of synthetic oversampling approaches, namely SMOTE and ADASYN.

The job of multiclass learning has been regarded as challenging for classification algorithms since multi-class classification generally yields worse results compared to binary scenarios [24]. The intricacy of this issue is heightened in the presence of unbalanced data since the borders between the classes exhibit significant overlap [25]. This study is designed to address the issue of imbalanced data in the field of depression prediction through the implementation of multiclass classification techniques.

The validation of training data is crucial in addressing the issue of overfitting in machine learning-based prediction [26]. The stratified k-fold cross-validation approach is often employed for performance validation [27]. This research stratified the samples based on the original distribution across multiple classes to enhance the robustness of the validation process.

To gauge the severity of depression there are currently many prevalent standard questionnaires that are used. Burn’s depression checklist (BDC) is, however, a trustworthy mood-measuring instrument to identify the existence of depression and provide an accurate rating of its severity [28]. The purpose of BDC is not treatment but rather an assessment of the need for further clinical and individual care [29]. BDC is widely used as a main screening tool for depression identification in a variety of medical institutions [30], universities [31, 32], and psychological counseling centers [33–39]. Moreover, organizations accommodate the screening process, where a score value is calculated by summing the response weights of the questions, and the score value is compared with the range value then the depression severity is estimated. This research used BDC as the depression screening tool.

Therefore, in the proposed FGP approach, initially, these relative features are considered as grouped and a score value by adding those features’ weights as a function of Individual Score (IS). The individual range value of each group is computed by interpreting a defined function called Individual Range (IR). Finally, the Individual Target (IT) function is used to find the target value for each group. Now, the corresponding target of each group is considered as an input feature for developing the machine learning model. In summary, the main contributions of this work are outlined as follows:

- The primary focus of this investigation is to create a machine learning based model to accurately predict the intensity of depressive disorder.
- This research aims to use the oversampling methods SMOTE and ADASYN to solve the problem of class imbalance.
- The application of a balanced dataset may reduce the biases and overfitting problem.
- To validate the FGP approach to enhance the accuracy and reduce the training time and required space.
- This work is meant to consider the issue of multiclass targets in the field of depression prediction.
- To make the validation procedure more reliable, this study used stratified samples based on equal distribution across different classes.

The paper is structured in the following manner. First, we briefly discuss the statistical data about depression disorder around the globe and the impact and effect that influence individuals’ lives with technological contribution regarding this issue that motivated us to do this research. After that, some similar work in this field, including their limitations and this study involvement has been provided. Later the methodology of the proposed FGP approach is explained with data acquisition, feature descriptions, imbalanced data handling technique, applied machine learning algorithms, and method of implementation. Subsequently, a comprehensive elucidation of the outcome and the used metrics for the assessment of performance is provided accompanied by a comparison analysis. Ultimately, the forthcoming direction of the investigation is expounded upon with the conclusion.

## Literature review

The literature review component was conducted in two distinct contexts, both of which are elaborated upon in this section. BDC consists of a comprehensive set of 25 questions that assess various symptoms associated with depression developed by Dr. David Burns, a distinguished American psychiatrist and adjunct professor emeritus in the Department of Psychiatry and Behavioral Sciences at the Stanford University School of Medicine [40]. The score value in this screening is determined by the summation of the weights assigned to each question’s response. Subsequently, the score value is compared with the range value provided by the BDC as outlined in Table 1, enabling an estimation of the severity of depression.

**Table 1:** BDC depression severity level

Level of depression	Total score
No Depression	0-5
Normal but unhappy	6-10
Mild depression	11-25
Moderate depression	26-50
Severe depression	51-75
Extreme depression	76-100

In this way, depression is detected in general. We named this depression detection approach as CDS method. The School of Medicine at Saint Louis University has a distinguished history of achieving high standards in medical school, graduate medical education, and graduate education. The institution employed the CDS approach and used BDC as a screening tool to evaluate the mental health status of its student population [30]. Also, certain universities [31, 32] initially use the CDS approach to know about students’ mental well-being, and depending on the obtained ratings, these institutions organize counseling sessions and arrange appropriate treatment for students if required. Many mental wellness centers and counseling centers [33–39] utilize the CDS technique in conjunction with BDC to evaluate the severity of depression in their patients to provide appropriate assistance. So, the transformation of this accessing technique to a machine learning-based data analysis may be able to increase the accuracy of the prediction and the required time for prediction may be reduced. The study [41] presented feature grouping, which utilizes five supervised machine learning algorithms (CNB, RF, KNN, DT, and GNB), to predict depression severity using BDC. Their investigation demonstrates that the CNB classifier achieves a maximum accuracy of 90.07%. Furthermore, the study did not address or consider the imbalanced dataset. In addition, there was no implementation of any methodological adjustment. Numerous studies have used machine learning algorithms to predict depression symptoms. A comparative analysis is given in Table 2.

**Table 2:** Machine learning based depression prediction

Related work	Depression screening method	Types of targets	Imbalance class handled	Applied algorithm
[42]	Kaggle dataset	Binary	No	KNN, SVM, LR, DT, RF, NB
[43]	BDC	Binary	Yes	SVM, DT, LGBM, Bagging, Gradient Boosting, AdaBoost
[44]	Depression Anxiety Stress Scale (DASS 21)	Multiclass	No	SVM
[45]	Kaggle dataset	Binary	Yes	RF, LR, NB
[46]	Beck Depression Inventory	Binary	Yes	RF
[47]	DASS 21	Multiclass	No	DT, NR, SVM, NB, LR
[48]	BDC	Binary	Yes	KNN, AdaBoost, XGBoost, Bagging, Weighted voting
[49]	Beck Depression Scale and DASS 21 Bangla Version	Binary	No	KNN, RF, SVM
[50]	DASS-21	Multiclass	No	DT, RF, NB, SVM, KNN
[41]	BDC	Multiclass	No	CNB, GNB, RF, DT, KNN

The previous studies that were carried out focused primarily on the use of a variety of screening methods for data collection from various types of participants. Various well-established classifiers were examined for their ability to predict depression. Most of the studies used binary classification or converted the severity into binary class. For multiclass targets, the distribution of the data is generally highly imbalanced. There was no premium resource provided to speed up the training, also no new data preprocessing method was introduced to get better results or accuracy. We implemented a machine learning based depression severity prediction approach for multiclass based target prediction along with stratified cross validation and class balancing technique. In addition, we proposed a Feature Group Partitioning (FGP) approach, here, corresponding features are accessed as individual groups, and the individual target for each group is estimated at the preprocessing phase. For evaluation purposes, we compared the performances between the CDS and FGP approaches.

## Methodology

The following subsections provide a detailed description of the study’s overall methodology.

### Data acquisition

**Fig. 1:**
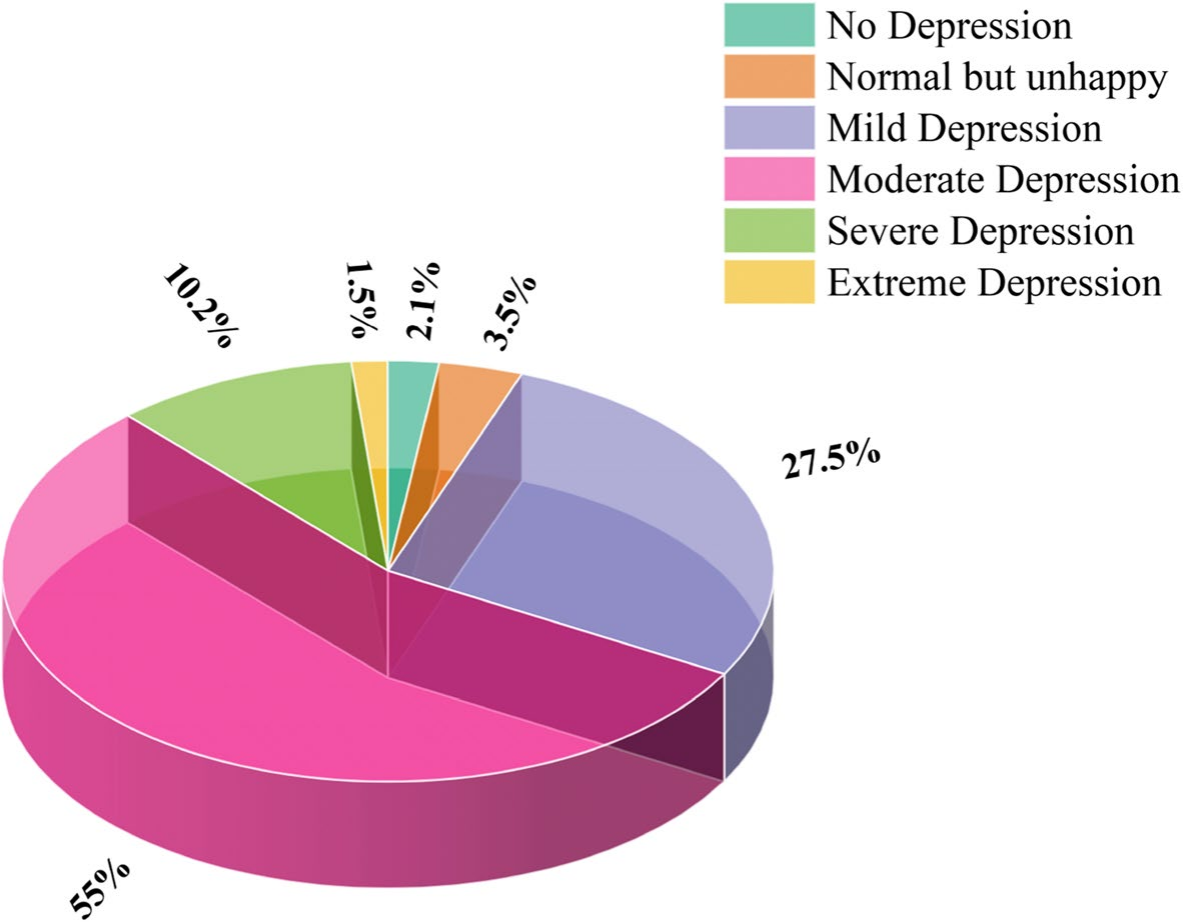
Distribution of depression severity among multiple classes

The dataset used in this study was obtained from Bangabandhu Sheikh Mujibur Rahman Science and Technology University, Gopalganj, a public university of higher education located in Bangladesh. A well-known depression severity screening self-reported questionnaire known as the Burns Depression Checklist (BDC) is utilized for the aim of data collection. BDC offers a dependable way of determining whether or not a person is depressed and determining the degree to which they are affected. The updated version of the BDC, which has 25 questions, is used in this study. Consent was taken from the authority to process the survey with the defined questionnaires. Our goal was to include students from all four years of study, both sexes and from distinguished ages. So that the result of depression levels has a realistic and impactful view on the education system and society. So, we performed individual interviews at the university campus to gather raw data. The dataset was acquired from the students of 34 departments that are organized into 7 faculties of the university. The student population includes individuals aged 19 to 28, with their academic progression ranging from the first to the fourth year of undergraduate studies. The dataset has a sufficient number of male and female individuals, with an equal ratio of 50:50. The dataset comprises the replies of 654 students. In this study, six tiers of depressive symptoms are analyzed. Figure 1 provides an overview of the prevalence and severity of depression throughout the whole sample. It is discernible that, among 654 students from the dataset 360 students are suffering from moderate levels of depression. This means 55% of students in our society are affiliated with depression, which is a very scary statistic to consider.

### Features description

This research makes consideration of a total of twenty-five predictor characteristics in addition to one target feature. Feature groups along with feature names and feature descriptions are presented in Table 3. The input features are retrieved from the updated version of BDC which are categorized into four groups. The thought and feelings group (TFG) consists of ten features that inquire about the participants’ current feelings and mental states, the next group, activities and personal relationships (APR), consists of seven features that inquire about the participants’ previous interactions and relationships with friends and family, and the physical symptoms group (PSG) is a group that conveys physical ailments with five features. The last group, the suicidal urges group (SUG) represents the suicidal desires of respondents and is comprised of the last three features. Each characteristic pertains to a different facet of the student’s mental health, each of which has some bearing on the student’s moods as well as the activities that they partake in daily. The type of features is categorical and ordinal. Each attribute is assigned a weight (W) among 0 (Not at all), 1 (Somewhat), 2 (Moderate), 3 (A lot), and 4 (Extremely). According to the prediction target (PT), the levels of depression are classified as follows: no depression, normal but unhappy, mild depression, moderate depression, severe depression, and extreme depression.

**Table 3:** Features for depression severity prediction

Feature names	Feature groups	Feature descriptions
F1	Thought and feelings group (TFG)	Feeling sad or down in the dumps
F2		Feeling unhappy or blue
F3		Crying spells or tearfulness
F4		Feeling discouraged
F5		Feeling hopeless
F6		Low self-esteem
F7		Feeling worthless or inadequate
F8		Guilt or shame
F9		Criticizing yourself or others
F10		Difficulty making decisions
F11	Activities and personal relationships (APR)	Loss of interest in family, friends, or colleagues
F12		Loneliness
F13		Spending less time with family or friends
F14		Loss of motivation
F15		Loss of interest in work or other activities
F16		Avoiding work or other activities
F17		Loss of pleasure or satisfaction in life
F18	Physical symptoms group (PSG)	Feeling tired
F19		Difficulty sleeping or sleeping too much
F20		Decreased or increased appetite
F21		Loss of interest in sex
F22		Worrying about your health
F23	Suicidal urges group (SUG)	Do you have any suicidal thoughts?
F24		Would you like to end your life?
F25		Do you have a plan for harming yourself?
Target variable	Prediction target (PT)	Depression severity

### Method of implementation

**Fig. 2:**
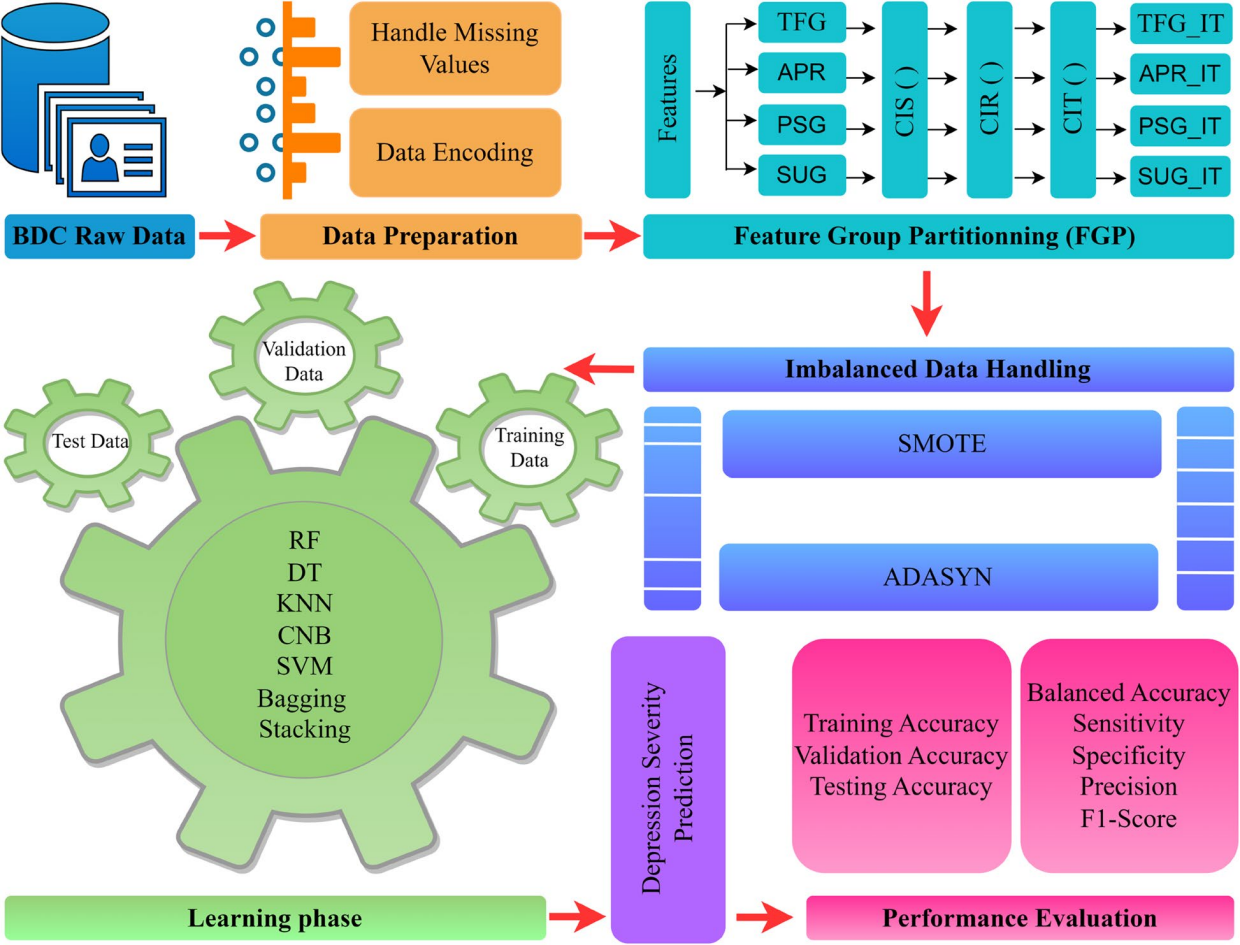
Feature group partitioning (FGP) based depression severity prediction approach

The steps that were followed to carry out this research are graphically represented in Fig. 2. The following sections will provide an in-depth description of each step.

#### BDC raw data

The BDC raw data set used by this analysis contains information on 654 students. It is structured by one target variable and twenty-five predictor variables with four distinct groups. A brief introduction of the features is presented in Table 4.

**Table 4:** Individual range (IR) with depression severity level (DSL)

Depression severity level (DSL)	Individual range (IR) for each FG				Prediction target range
	TFG-IR	APR-IR	PSG-IR	SUG-IR	PTR
No depression range (NDR)	0 to 2	0 to1	0 to 1	0 to 1	0 to 5
Normal but unhappy range (NUR)	3 to 4	2 to 3	2 to 2	2 to 2	6 to 10
Mild depression range (MDR)	5 to 10	4 to 7	3 to 5	3 to 3	11 to 25
Moderate- depression range (MDR)	11 to 20	8 to 14	6 to 10	4 to 6	26 to 50
Severe depression range (SDR)	21 to 30	15 to 21	11 to 15	7 to 9	51 to 75
Extreme depression range (EDR)	31 to 40	22 to 28	16 to 20	10 to 12	76 to 100

#### Data preparation

The data was prepared in two ways. Firstly, the values that were missing in the data set were addressed. To achieve this objective, we conducted a tally of the null values included in the dataset and subsequently eliminated the corresponding rows. As we mentioned before the data was collected through face-to-face interviews. Consequently, the occurrence of missing data was minimal. Secondly, we utilized the Label Encoder technique for data preprocessing. The features type was categorical and the possible values were string for all. The process of label encoding involves the conversion of labels into a numerical format that is easily interpreted by a machine.

#### Feature group partitioning (FGP)

Following the completion of data preparation, the encoded characteristics were employed in the FGP algorithm. This research considers TFG, APR, PSG, and SUG as individual groups. Initially, distinct groups contain their features. The three primary functions highlighted in FGP are CIS (), CIR (), and CIT (). In this context, CIS stands for Compute Individual Score, CIR stands for Compute Individual Range, and CIT means Compute Individual Target. The CIS () function is used to calculate the score value and the CIR () function converts the range value. Finally, the CIT () function finds the target for each group. Individually all groups performed these three functions and found individual targets as TFG_IT, APR_IT, PSG_IT, and SUG_IT CIS ( ). This method generates an individual score value for each group. Some variables were declared and initialized at the outset of this function. In this context, “N” refers to the total number of features. The feature weights assigned by each participant were stored in the variable W[N]. The aggregate number of features contained inside each group serves as an indicator of the threshold value for that particular group. After that, we sum the W for each group up to the group threshold, and that number becomes the IS value for that particular group. As initialized the variables *N*= 25 and the threshold values are TFG threshold (TFGth)10, APR threshold (APRth)7, PSG threshold (PSGth)5, and SUG threshold (SUGth) 3.

$$\begin{aligned}{} & {} \text {CIS (W[N], TFGth, APRth, PSGth, SUGth)}\\{} & {} \qquad \qquad \qquad \qquad \qquad \{ \\{} & {} \qquad \qquad \qquad \qquad \qquad \qquad \qquad \begin{array}{l} \text {TFG-IS} = \sum \limits _{i=1}^{\text {TFGth}} W[i] \\ \text {APR-IS} = \sum \limits _{i=1}^{\text {APRth}} W[i] \\ \text {PSG-IS} = \sum \limits _{i=1}^{\text {PSGth}} W[i] \\ \text {SUG-IS} = \sum \limits _{i=1}^{\text {SUGth}} W[i] \end{array}\\{} & {} \qquad \qquad \qquad \qquad \qquad \} \end{aligned}$$

To find the prediction target we calculate the Prediction target score (PTS) based on Eq. (1).

1 $$\begin{aligned} \text {PTS} = \text {TFG-IS + APR-IS + PSG-IS + SUG-IS} \end{aligned}$$

CIR () The purpose of this method is to generate an outline of the Depression Severity Level (DSL) by extracting the individual range values for each $\textrm{FG}$ exclusively. Individual range means the limit of each group to be considered for the DSL. Here, the weight threshold (Wth) considers the highest possible value for each feature and this value is initialized as 4. The score threshold (Sth) represents the highest score for each FG and this value is calculated by multiplying the $\textrm{Wth}$ with $\textrm{F}[{n}]$. For No Depression Range (NDR), the starting limit is 0 and the upper limit of the range is calculated by multiplying the Sth with a constant value a. We consider a total of 5 constant values including *a*, *b*, *c*, *d*, *e*, and the value of this constant is calculated by adapting the original BDC depression severity level. The Normal but Unhappy Range (NUR) lower limit is the higher limit of NDR and the upper limit is calculated by multiplying the $\textrm{Sth}$ with the $\textrm{b}$ constant. Finally, the Extreme Depression Range (EDR) higher limit is defined by the value of Sth. The considerations are the number of $F G, {n}=4$, Number of features in each $F G, F[{n}]=\{10,7,5,3)$.

$$\begin{aligned}{} & {} \text {CIR}(\text {F}[n],\ \text {W th, Sth})\\{} & {} \{\text {for i 1 to}\ {n}\\{} & {} \qquad \text {Score threshold, Sth[i]} = \text {F[i]}^{*}\ \text {Wth}\\{} & {} \qquad \text {No Depression Range, NDR[i]} = \text {round}(a *\text {Sth}[i])]\qquad [\text {a} = 0.05]\\{} & {} \qquad \text {Normal but Unhappy Range, NUR[i]} = \text {round}\ (\ b *\text {Sth}[i]\ )\ [\text {b} = 0.1]\\{} & {} \qquad \text {Mild Depression Range, MDR[i]} = \text {round}(c *\text {Sth}[i])[\text {c} = 0.25]\\{} & {} \qquad \text {Moderate-depression Range MR[i]} = \text {round}\ (d *\text {Sth}[i])[\text {d} = 0.5]\\{} & {} \qquad \text {Severe Depression Range, SDR[i]} = \text {round}\ (e *\text {Sth}[i])[\text {e} = 0.75]\\{} & {} \qquad \text {Extreme Depression Range, EDR[i]} = \text {Sth}[i]\\{} & {} \} \end{aligned}$$

Now, the individual ranges of each group are given in Table 4. The prediction target range (PTR) is calculated by the BDC depression severity level.

The activity of the ‘CIT ()’ function 3 is mainly to convert the features of a group into one target feature. It compared the IS with IR and finalized the IT. There IS value for distinct groups like TFG, APR, PSG, and SUG is considered and compared based on the range like TFG-IR, APR-IR, PSG-IR, and SUG-IR. Each group makes use of this function separately. For instance, in feature group TFG, if TFG-IS is less than or equal to NDR of TFG_IR then the TFG-IT is considered as ’no depression’ else if TFG-IS is less than or equal to NUR of TFG_IR then the target value will assign ’normal but unhappy’. In this way, mild, moderate, severe, and extreme levels of depression are calculated by comparing the value of MDR, MR, SDR, and EDR of TFG_IR with TFG-IS. Remain groups also follow the same pathway to find the APR_IT, PSG_IT, and SUG_IT. Besides, the prediction target $(\textrm{PT})$ is calculated by comparing it with PTS and PTR.

$$\begin{aligned}{} & {} \text {CIT ()} \{ \\{} & {} \text {if IS}\ {<=}\ \text {NDR then IT} = \text {No depression}\\{} & {} \text {else if IS}\ {<=}\ \text {NUR then IT} = \text {Normal but unhappy}\\{} & {} \text {else if IS}\ {<=}\ \text {MDR then IT} = \text {Mild depression}\\{} & {} \text {else if IS}\ {<=}\ \text {MR then IT} = \text {Moderate depression}\\{} & {} \text {else if IS}\ {<=}\ \text {SDR then IT} = \text {Severe depression}\\{} & {} \text {else if IS}\ {<=}\ \text {EDR then IT} = \text {Extreme depression}\\{} & {} \quad \} \end{aligned}$$

#### Imbalance data handling

When a classifier is trained with data that is not evenly distributed, it produces predictions that are not just biased but also incorrect. The proportion of individuals in the training datasets who have no signs of depression is 2%, the percentage who are normal but unhappy is 4%, those who are mildly depressed are 28%, moderately depressed are 55%, severely depressed are 11%, and the highly depressed rate is just 1%. Sampling is a method for dealing with imbalanced data. Under-sampling and oversampling are two forms of sampling. Undersampling is a technique where the possibility of losing important information is a big flaw, and this is addressed and counteracted by using the oversampling technique. The term “oversampling” refers to increasing the number of minority-class samples to balance with the majority class. Because the training datasets include a significant amount of class imbalance, we employed the widely-used benchmark oversampling algorithms SMOTE [51] and ADASYN [52] to the data to rectify the imbalanced classes. The number of samples generated before and after running SMOTE and ADASYN is displayed in Table 5.

**Table 5:** The scenario of handling imbalance data

Depression among targets	No depression	Normal but unhappy	Mild depression	Moderate depression	Severe depression	Extreme depression	Total samples
CDS Approach	14	23	180	360	67	10	654
FGP Approach	14	23	180	360	67	10	654
FGP with SMOTE	360	360	360	360	360	360	2160
FGP with ADASYN	360	360	360	360	360	360	2160

##### Synthetic Minority Oversampling Technique (SMOTE)

In 2002, Chawla et al. developed the SMOTE regular approach [51], where the minority class includes synthetic minority class samples that are uniformly dispersed around the original positive cases. By conducting its operations in feature space, SMOTE produces artificial samples from the underrepresented group. First, SMOTE locates the k closest neighbors of the minority group’s data points. Then, it generates a new point somewhere in a completely random position relative to its neighbors. These fresh dots stand in for fabricated statistics that make up the marginalized group. Finally, it will keep producing fresh data until the problem with the data imbalance has been fixed. The feature vector of the under-investigation minority class sample is denoted by $f_i$, and $f_{\text{ near } }$ is one of the K-nearest neighbors of $f_i$. Equation (2) is a representation that is used for the newly created synthetic sample $\textrm{f}_{\text {new}}$.

2 $$\begin{aligned} f_{\text {new}}=f_i+\left( f_i-f_{\text {near}}\right) \times R \end{aligned}$$

*R* is a random number between 0 and 1 in this scenario.

##### ADASYN

ADASYN is often cited as the first to create synthetically derived algorithms like SMOTE. By adaptively shifting the categorization decision boundary toward the challenging samples, ADASYN mitigated the bias generated by the class imbalance [52]. Overall, ADASYN makes use of nearest neighbors to automatically provide more artificially generated information of minority class samples weighted according to their distributions. First, it determines the level of inequality between the classes. The amount of synthetic data examples that need to be created for the minority class is then computed. After that, choose a random minority data sample among $\textrm{K}$ nearest neighbors for each minority class data sample, and then construct the synthetic data $f_{\text{ new } }$, using Eq. (3).

3 $$\begin{aligned} f_{\text {new}}=f_i+\left( \textrm{fz}_{\textrm{i}}-f_i\right) \times \lambda \end{aligned}$$

Here, $f_i$ is an example of data from the minority class, $fz_i$ - $f_i$ represents the difference vector in *n* dimensional spaces, and a random number: $\lambda \in [0,1]$.

#### Learning phase

We initially applied five supervised machine-learning algorithms that are clinically applicable to analyze depression types mental health disorders [16–18]. The machine learning algorithms utilized in this study include Decision Trees (DT), Random Forests (RF), Support Vector Machines (SVM), Categorical Naive Bayes (CNB), and K-nearest neighbors (KNN).

##### Decision Tree

The binary splitting criterion of a decision tree is extended to many classes to make the decision tree suitable for use in multiclass classification issues [53]. The Gini index, which determines the degree to which a node is impure, is used as a criterion for this analysis. Gini criteria for multiclass classification are given in Eq. (4) [54].

4 $$\begin{aligned} \text {Gini}(\textrm{D}) = 1-\sum \limits _{k=1}^K(\textrm{p}[k] \times P[k]) \end{aligned}$$

Here, Gini(D) is the Gini impurity of node, *K* is the number of classes, and *P*[*k*] is the proportion of instances in class *k* at node D. Finding the feature split that results in the lowest possible Gini index at each node is the objective of using a decision tree. For each node, the optimal split is determined to be the one that yields a Gini index that is lower than the others.

##### Random Forest

The Random Forest Classifier is a highly effective machine learning method utilized for multiclass prediction assignments. It is a strategy known as an ensemble that combines several different decision trees to produce precise and reliable forecasts. In this approach, individual trees are trained autonomously using a randomly selected subset of the training data and a randomly selected subset of the features. The incorporation of randomness in the model aids in mitigating overfitting and enhancing the model’s ability to generalize. In this particular investigation, the dataset featured multiclass as the aim; more specifically, here, every decision tree in the forest independently predicts a class for a given input. The ultimate prediction is arrived at by using a voting system known as majority voting [55]. In this method, the class that receives the greatest number of votes from all of the trees is chosen to be the predicted class. To ascertain the number of trees within the forest, the parameter $\textrm{n}_{-}$estimators are established at a value of 100. The parameter min_samples_split was modified to a value of 2 to regulate the growth of the tree. Similarly, the parameters max depth and min samples leaf are assigned the values of none and 1, respectively. The primary measure employed to assess the effectiveness of a split is the Gini impurity criterion.

##### KNN

The K-nearest neighbors (KNN) algorithm possesses the inherent capability to effectively address multiclass classification tasks without necessitating any alterations or modifications [56]. It is a form of instance-based learning in which the categorization of a new data point is decided by the class that holds the majority of its k-nearest neighbors. To carry out the implementation, we establish the parameter n_neighbors with a value of 3. The estimation of similarity between instances is achieved through the utilization of distance metrics. The parameter metric is specified as ’Minkowski’ to calculate the distances between the data points, mathematically represented by Eq. (5) [57].

5 $$\begin{aligned} D(X, Y)=\left( \sum \limits _{i=1}^{n}\left| x[i]-y[i]\right| ^{p}\right) ^{\frac{1}{p}} \end{aligned}$$

Here, *p* is the order of the Minkowski distance and the data points are $\textrm{X}(x[1], x[2], x[3], \ldots , x[n])$ and $\textrm{Y}(y [1], y [2], y [3], \ldots ,y[n])$. After that, we pick the *k* data points that are closest to the new and have the shortest distances between them. After that, we tally up the instances of each class among the *k* closest neighbors and give a new designation of the class that appears the greatest number of times among those neighbors.

##### Categorical Naive Bayes

The Categorical Naive Bayes classifier is an effective probabilistic machine learning technique for multiclass prediction. It is a method that builds on Bayes’ theorem and assumes feature independence within a given class. It establishes a connection between the prior probability of a class and the probability of the data given the class, as well as the conditional probability of a class given the data. The most likely probability turns out to be predicted. In this study, we set the alpha value as 1 and force_alpha and fit_prior as true for the CNB classifier.

##### Support Vector Machine

Support Vector Machines (SVM) were initially devised for binary classification. However, there exist methodologies to expand its applicability to address multiclass situations. The main objective of Support Vector Machines (SVM) is to identify the optimal hyperplane that effectively separates the data points into two distinct classes by maximizing the margin [58]. SVM can employ various kernel functions, such as linear, polynomial, and radial basis functions, to effectively capture intricate and nonlinear associations within the dataset. The present investigation employed a linear kernel with a degree of 3 and a gamma value of ’scale’. SVM possesses the capability to ensure predictive performance, therefore making it widely employed across mental health domains.

##### Bagging

The concept of bagging was developed by leveraging the principles of bootstrapping and aggregating. The Bagging classifier involves the construction of bootstrap datasets from the training dataset. Subsequently, each of these bootstrap datasets is employed to train distinct classifiers. Lastly, the outcomes of these classifiers are combined to provide the ultimate forecast. Misleading training objects are frequently circumvented in the bootstrap dataset. According to [59] the performance of many classifiers is generally superior to that of a single classifier when aggregated. The Bagging classifier, by combining both of these properties, frequently demonstrates greater performance compared to alternative classifiers. The present study has conducted the Bagging technique on Multilayer Perceptron (MLP) classifiers. The MLPClassifier is configured with a hidden_layer_sizes parameter of 100 and a max_iter parameter of 1000. The value assigned to the parameter n_estimators is 10 throughout the bagging process.

##### Stacking

Stacking ensemble learning is a very effective methodology utilized in multiclass classification tasks, which capitalizes on the collective capabilities of many models to augment predicted precision and resilience [60] The process of stacking entails the training of several base models using the identical dataset, followed by the training of a meta-model that amalgamates the predictions generated by the base models. The utilization of stacking methodology might yield valuable insights regarding the significance of many base models and their respective contributions towards the ultimate forecast. The meta-model acquires the optimal approach for weighting and utilizing the predictions generated by the basic models to arrive at the ultimate categorization determination. We identify the top four algorithms based on their accuracy in the FGP method of our study. These algorithms are subsequently used to construct a heterogeneous ensemble model known as stacking. The base learners adopted in our stacking model included DT, RF, CNB, and KNN, while the meta-learner was RF.

#### Depression severity

Prediction and Performance Evaluation We have evaluated the performances of four methods including the existing depression assessment method CDS, the proposed FGP method, FGP with data balanced by SMOTE, and the FGP method with ADASYN. Firstly, to justify the overfitting problem we analyze the result from the perspective of training, validation, and testing accuracy. After that, to estimate the prediction performance we review the evaluation parameters such as balanced accuracy, sensitivity, specificity, precision, and f1-score using the following Eqs. (6)–(10):

6 $$\begin{aligned} \text {Balanced accuracy} = \frac{\text {Sensitivity} + \text {Specificity}}{2} \end{aligned}$$

7 $$\begin{aligned} \text {Sensitivity} = \frac{T P}{T P+F N} \end{aligned}$$

8 $$\begin{aligned} \text{ Specificity } = \frac{T N}{T N+F P} \end{aligned}$$

9 $$\begin{aligned} \text {Precision} = \frac{T P}{T P+F P} \end{aligned}$$

10 $$\begin{aligned} \text {F1 Score} = \frac{2 \times T P}{2 \times T P+F P+F N} \end{aligned}$$

Here,

- TP: TP is referred to as an assessment when an individual who is experiencing depression is correctly identified as depressed by the ML algorithm.
- TN: TN is referred to as an assessment when an ML algorithm accurately predicts an individual without depression as not depressed.
- FP: FP is referred to as an assessment when a machine learning algorithm incorrectly predicts an individual without depression as depressed.
- FN: FN is referred to as an assessment when an individual who has been diagnosed with depression is incorrectly classified as not depressed by the classifier.

Secondly, we simulate each approach and each classifier to get the required Training Time (TT). Finally, the space complexity of the FGP method is evaluated.

## Result analysis and discussion

This section provides an overview of the experimental setting and the assessment criteria used to assess the effectiveness of the FGP approach. Our experimental investigation focuses on evaluating the performance of the FGP technique, specifically concerning the performance of the CDS method. In addition, we employed class balancing techniques, particularly SMOTE and ADASYN on the dataset and implemented the FGP method on this balanced dataset. Synthetic sampling might produce unrealistic or noisy data, which can cause over-fitting problems. Moreover, it can also introduce computational complexity, thereby impacting the training time of machine learning models. However, this analysis demonstrated the enhancement of model performance in Table 8, also the overfitting situation was illustrated remarkably in Fig. 4 for the balanced dataset.

**Fig. 3:**
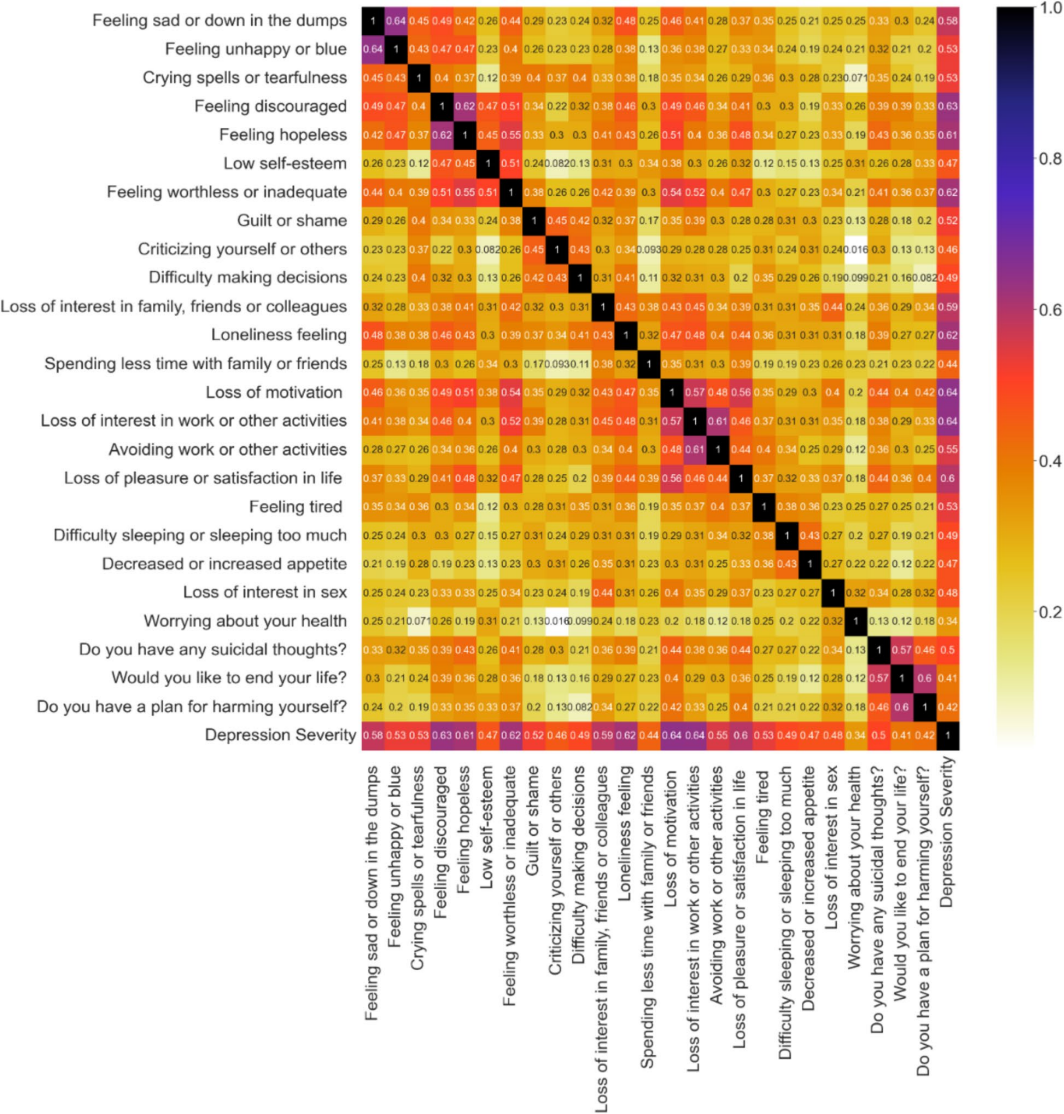
Coefficient values of pearson correlation analysis

We conducted a statistical parametric analysis on our dataset to determine the magnitude and direction of the association between the input and the target features. The Pearson correlation coefficient values are provided in Fig. 3. However, the outcome does not exhibit any significant correlation among the variables.

This research used the Python programming language and the sci-kit learn module [61] for implementation purposes. Before implementing a machine learning method, it is customary to partition the dataset into separate training, validation, and testing sets. To facilitate the training process, a subset comprising 60% of the whole dataset was utilized. Additionally, a stratified 10-fold cross-validation technique was employed on a 20% subset to make sure the models were not overfitted. Subsequently, we conducted testing on the remaining 20% of the data to determine the performance of the models. Table 6 demonstrates the discrimination among training, validation, and testing accuracy for CDS and FGP approaches with imbalanced data.

**Table 6:** Representation of training, validation and testing accuracy of CDS and FGP approach with imbalanced dataset

Applied algorithm		RF	DT	KNN	CNB	SVM	Bagging	Stacking
CDS approach	Training Accuracy	100	100	92.16	88.53	99.43	98.47	100
	Validation Accuracy	85.29	71.89	83.95	83.78	83.69	76.68	84.15
	Testing Accuracy	86.26	74.05	88.55	82.44	81.61	79.39	85.79
FGP approach	Training Accuracy	94.26	94.26	91.21	91.01	88.91	92.93	94.26
	Validation Accuracy	88.32	87.94	88.72	88.91	87.39	89.47	87.94
	Testing Accuracy	87.79	87.79	89.31	85.08	87.02	89.31	87.79

The average difference between the training and validation accuracy is 15 percent for the CDS approach. Moreover, the testing accuracy is 14 percent less than the training accuracy. This enormous difference between the training, validation, and testing accuracy represents the overfitting and abnormality of the data structure for the CDS. Whereas, the analysis of training, validation, and testing accuracy for the FGP approach reduces the gap between them to a minimum amount. Here, the variance for the training and validation is 4 percent and for the testing set the variance is 4.5 percent. As the FGP approach reduces the number of features, so, the variance has been optimized tremendously.

**Fig. 4:**
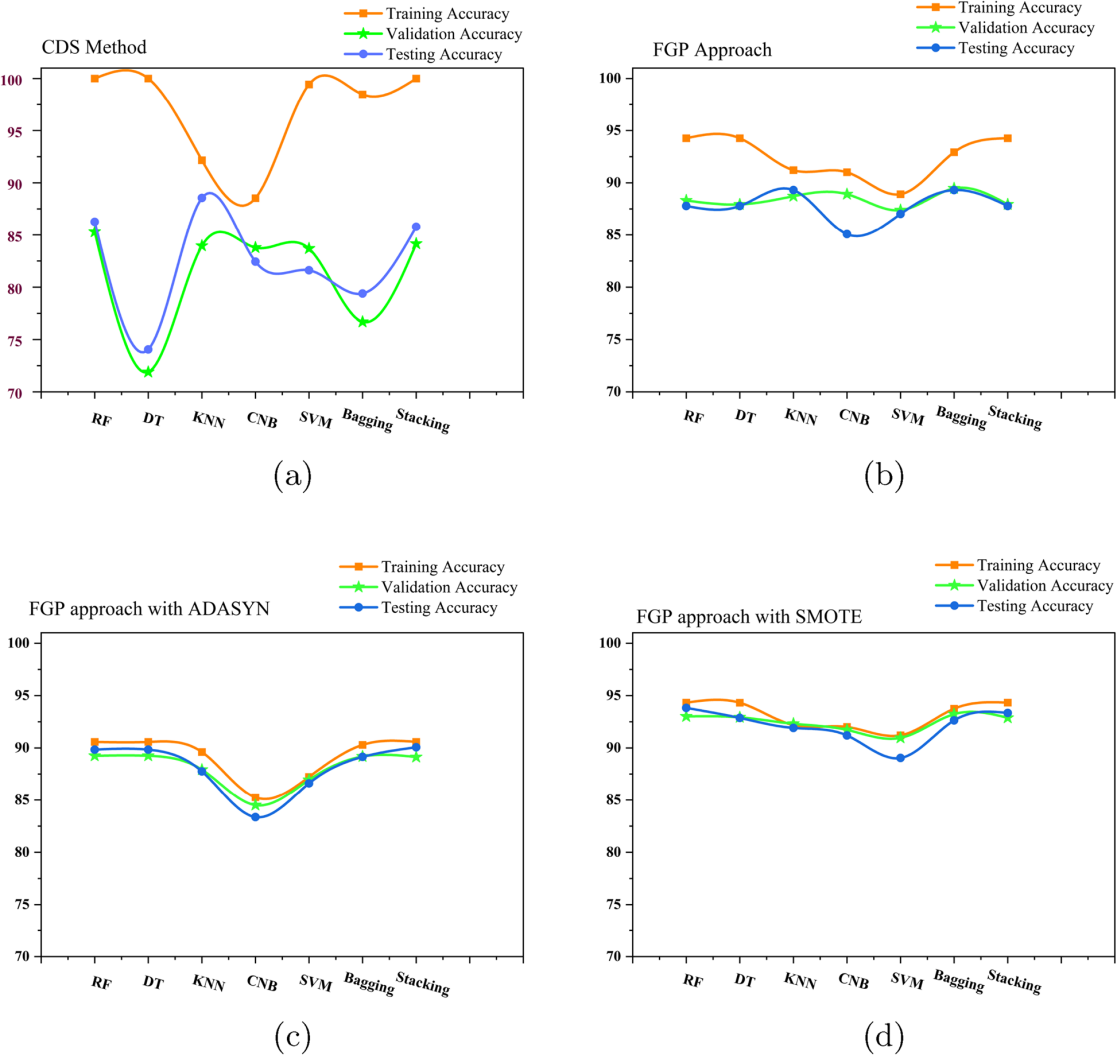
**a** Training, validation, and testing accuracy for CDS approach. **b** Training, validation, and testing accuracy for FGP approach. **c** Training, validation, and testing accuracy for FGP approach with ADASYN. **d** Training, validation, and testing accuracy for FGP approach with SMOTE

After that, we balanced the dataset through oversampling techniques SMOTE and ADASYN to analyze the training, validation, and testing accuracy of the FGP approach and it showed a remarkable amount of reduced variance as presented in Table 7. On average, the training and validation accuracy gap for the FGP method with the SMOTE is only 0.7, and for the ADASYN dataset is 1.2 percent. The training testing gap for the ADASYN dataset is only 1.1 and 1.7 percent for the SMOTE dataset. The highest accuracy is produced by the RF classifier including 94.33 as training, 92.01 as validation, and 93.06 as testing accuracy. A graphical depiction of training, validation, and testing accuracy for CDS, FGP, FGP with SMOTE, and FGP with ADASYN is given in Fig. 4. It is presented that the disparity among training, validation, and testing accuracy is reduced significantly when applying SMOTE with FGP.

**Table 7:** Representation of training, validation and testing accuracy of FGP approach with a balanced dataset

Applied algorithm		RF	DT	KNN	CNB	SVM	Bagging	Stacking
FGP with SMOTE	Training Accuracy	94.33	94.32	92.19	92.01	91.21	93.75	94.33
	Validation Accuracy	93.01	92.93	92.31	91.72	90.97	93.23	92.88
	Testing Accuracy	93.06	92.13	91.21	90.51	88.43	91.91	92.59
FGP with ADASYN	Training Accuracy	90.58	90.58	89.61	85.26	87.23	90.29	90.58
	Validation Accuracy	89.25	89.25	87.92	84.51	86.94	89.19	89.13
	Testing Accuracy	89.84	89.84	87.76	83.37	86.61	89.15	90.07

Imbalanced data needs more concern than balanced data to evaluate the performance of machine learning algorithms. As the nature of the primary dataset used by this study is imbalanced, we considered balanced accuracy, specificity, sensitivity, precision, and f1 score parameters to evaluate the performance of the classifier used in different approaches. We used the weighted average score for specificity, sensitivity, precision, and f1 score. The comparative analysis through performance evaluation parameters is given in Table 8.

**Table 8:** Comparative performance analysis of applied algorithms for four approaches

Method name	Applied algorithms	Balanced accuracy	Sensitivity	Specificity	Precision	F1-score
FGP with SMOTE	RF	92.31	92.36	98.41	92.64	92.34
	DT	92.06	92.13	98.36	92.45	92.11
	KNN	90.97	91.21	98.15	91.47	91.17
	CNB	90.55	90.51	98.06	90.81	90.48
	SVM	88.56	88.43	97.65	88.66	88.51
	Bagging	91.89	91.9	98.33	92.11	91.89
	Stacking	92.54	92.59	98.46	92.83	92.58
FGP with ADASYN	RF	90.51	90.07	98.06	90.61	90.07
	DT	90.18	89.84	97.99	90.38	89.86
	KNN	88.39	87.76	97.62	89.29	87.68
	CNB	82.97	83.37	96.66	83.91	83.11
	SVM	86.84	86.61	97.42	87.34	86.74
	Bagging	89.49	89.15	97.87	89.79	89.19
	Stacking	90.51	90.07	98.06	90.61	90.07
FGP approach	RF	80.53	87.79	93.32	88.89	87.35
	DT	80.53	87.79	93.32	88.89	87.35
	KNN	81.07	89.31	93.69	90.05	88.79
	CNB	79.12	90.08	93.78	90.73	88.96
	SVM	79.49	87.02	88.29	88.01	86.88
	Bagging	77.7	89.31	92.76	89.91	88.18
	Stacking	80.53	87.79	93.32	88.89	87.35
CDS approach	RF	76.29	87.02	90.06	88.12	86.04
	DT	74.04	74.05	83.24	75.06	74.24
	KNN	80.97	88.55	94.37	90.47	89.16
	CNB	76.11	82.44	90.93	84.33	82.73
	SVM	79.05	91.61	85.47	90.93	91.11
	Bagging	65.54	79.39	81.32	76.53	77.37
	Stacking	70.84	76.34	87.47	79.97	75.79

Because each statistic offers a distinctive perspective on a different aspect of a model’s performance, these metrics are essential instruments for evaluating and contrasting the various models. Balanced accuracy offers a comprehensive assessment of a model’s ability to accurately classify cases across all classes, ensuring a fair evaluation even in the presence of imbalanced class sizes. It is observed from Table 8, that the SVM classifier achieves a balanced accuracy of 79.05 when using the CDS method, 79.49 when using the FGP method, increased to 86.84 when using ADASYN, and the performance further increases to 88.56 when using the SMOTE method. For CDS, the CNB classifier produces a balanced accuracy of 76.11, for FGP, it produces 79.12, for ADASYN, it produces 82.97, and for SMOTE, the accuracy increases to 90.55. Similarly, the KNN classifier and bagging methods attained the highest balanced accuracy for the FGP with the SMOTE approach, with values of 90.97 and 91.89 respectively, surpassing the other three approaches.

Due to its capacity to gather intricate data and offer a lucid representation and comprehension of the decision-making process with a high level of interpretability, DT is highly suitable for modeling the interactions among the several factors that contribute to depression symptoms. In this analysis, DT generates 92.06 percent accuracy for the dataset that is balanced by SMOTE, and 90.18 percent for ADASYN, whereas for imbalanced data it generates 80.53 and 74.04 percent for FGP and CDS approaches, respectively.

For the RF classifier, we aggregate the predictions of 100 DT classifiers to create a more robust model that has provided the capability to predict more precisely new data. The balanced dataset yielded the maximum accuracy rate of 92.31 percent for RF. We found that stacking produces the highest accuracy for FGP with SMOTE and the value is 92.54 as it efficiently leverages the advantageous qualities of four basic models to enhance overall performance. The application of various class balancing approaches in conjunction with FGP has resulted in a significant improvement in the accuracies of all the classifiers. Especially SMOTE class balancing technique generates maximum accuracies for our dataset.

In addition, for gauging a model’s efficacy, sensitivity and specificity are crucial metrics to consider. A more sensitive model is better able to detect participants who are depressed, while a more specific model is better able to distinguish between participants who are and are not depressed. In FGP with SMOTE method, the stacking algorithm produces the highest sensitivity, specificity, precision, and f1-score and the values are 92.59, 98.46, 92.83, and 92.58 respectively. Ultimately, after evaluating the performance of all classifiers using four different approaches, it can be confidently said that the SMOTE oversampling technique, combined with the FGP approach, has significantly enhanced all performance indicators and in addition, the stacking classifier has outperformed best.

**Fig. 5:**
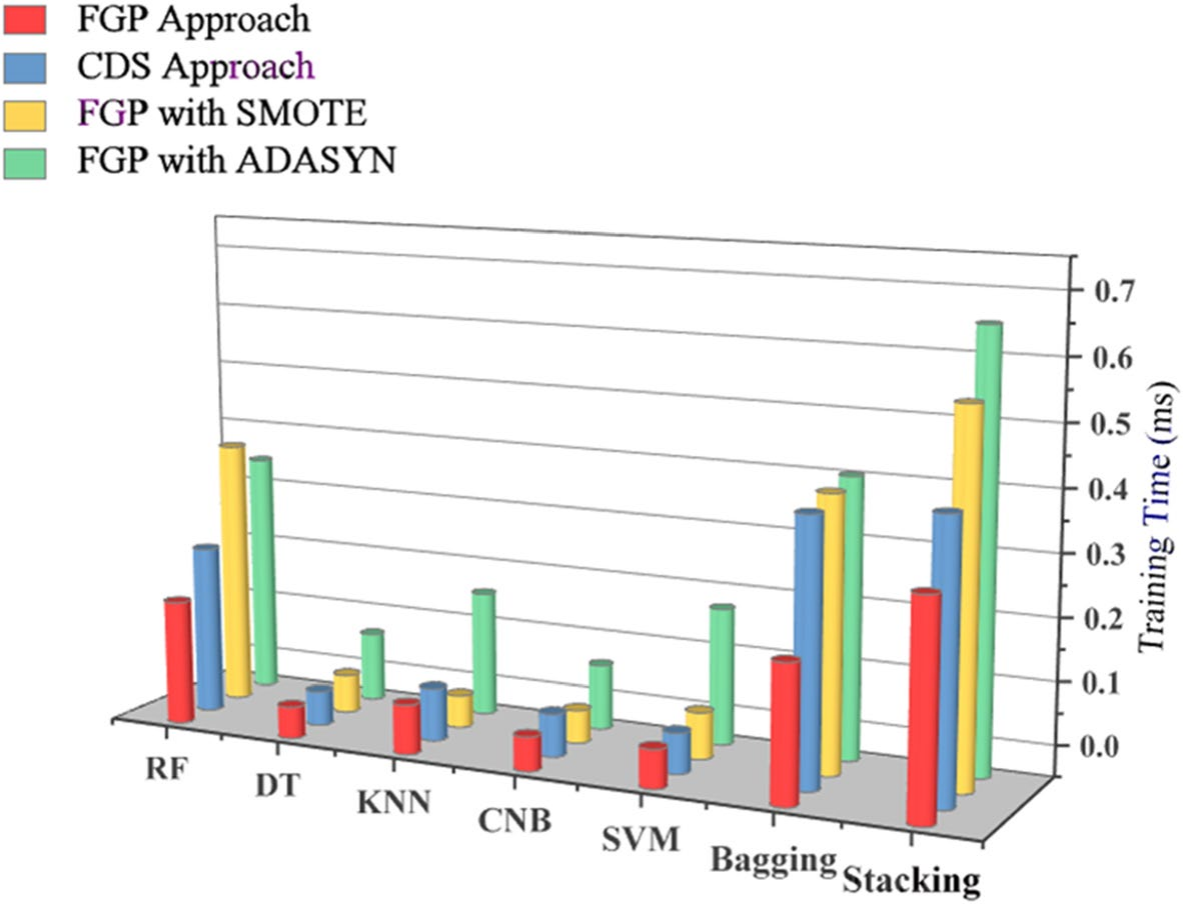
Training time analysis for all approaches

Figure 5 illustrates the comprehensive comparison of TT across all the approaches included in this study. Initially, the amount of data was 654 and we examined the time needed for both the CDS and FGP techniques. Our analysis revealed that all the classifiers utilized in the FGP technique exhibited shorter processing times compared to CDS. Subsequently, we augmented the data set by oversampling the minority classes and performed a second estimation of the TT for FGP using SMOTE and ADASYN. The findings indicate that the TT exhibited an increase as the data volume increased for the FGP. In addition, SMOTE requires less time than the ADASYN oversampled dataset, except for the RF classifier. Although KNN has the lowest TT, stacking demonstrates the highest performance for the SMOTE dataset based on accuracy assessment. Therefore, it is worth considering the trade-off between accuracy and time requirements.

The amount of memory an algorithm needs to process an input varies depending on its size, and this variable is referred to as the algorithm’s space complexity. When working with huge datasets on machines with limited resources, it is vital to keep the space complexity as low as possible. The CDS approach has a space complexity of $\mathcal {O}(N \times S)$, while the FGP approach has a space complexity of $\mathcal {O}(n \times S)$. Here, S represents the number of training samples. The FGP strategy in this study demonstrated a 16% reduction in memory usage compared to the CDS approach, achieved by feature grouping by decreasing the number of features.

## Conclusion and future work

In the community that studies machine learning, the subject of class imbalance is still considered an open research question. In this work, we have performed comparative research based on the different supervised machine learning algorithms, bagging, and stacking approaches to predict the severity of depression among Bangladeshi students. In particular, we addressed the issue of class imbalance and used SMOTE and ADASYN methods in our solution development. In addition, we used the FGP approach during the data preparation phase. The stacking approach generates the highest accuracy with amazing sensitivity, specificity, precision, and f1-Score. This research contribution to FGP with the SMOTE method has offered superior accuracy than any of the other classifiers. The problem of overfitting was also identified through the use of stratified cross-validation in the result analysis. The overall performance of the proposed FGP approach has been validated by comparisons of training times as well as analyses of space complexity. In the future, we are going to look at the possibility of performing further research on hyperparameter adjustment of the classifiers. Various techniques for feature selection and the application of explainable AI can yield the value of features and effectively identify the features associated with depression symptoms for future implementation.

## Data Availability

The data that support the findings of this study can be obtained from corresponding authors upon reasonable request.
